# 离子色谱电渗析技术的研究进展

**DOI:** 10.3724/SP.J.1123.2020.07016

**Published:** 2021-02-08

**Authors:** Bingcheng YANG, Zongying LI

**Affiliations:** 华东理工大学药学院, 上海 200237; School of Pharmacy, East-China University of Science and Technology, Shanghai 200237, China; 华东理工大学药学院, 上海 200237; School of Pharmacy, East-China University of Science and Technology, Shanghai 200237, China

**Keywords:** 电渗析技术, 样品前处理, 电致淋洗液发生器, 电致膜抑制器, electrodialysis technique, sample preparation, electrodialytic eluent generator (EDG), electrodialytic membrane suppressor

## Abstract

电渗析器件通常定义为在电场作用下操纵离子从一种溶液穿过离子交换膜迁移到另外一种溶液的一种设备。它可以通过电解水产生氢离子或氢氧根离子,从而用于离子色谱系统的淋洗液在线制备、抑制或检测。相较于人工配制淋洗液或再生液,电渗析技术具有绿色、高效、纯度高、自动化程度高等优势。因此基于电渗析器件的离子色谱系统应用范围越来越大。该文简要评述了近几年该器件的研究进展,具体包括电致淋洗液发生器、电致膜抑制器和电渗析样品前处理器。

## Abstract (en)

An electrodialysis device is used to transport salt ions from one solution to another through an ion-exchange membrane under the influence of an applied electric potential. They can produce hydronium or hydroxide ions via electrodialysis water, which can be used for online preparation of eluents or suppression or detection in ion chromatography (IC). As opposed to the manual preparation of eluents or regenerant solutions, electrodialysis devices have the advantages such as green, efficient and high purity. Thus, IC systems equipped with electrodialysis devices dominate the majority uses of IC system, and more importantly, find widespread application. The present thesis briefly summarizes the latest developments in this area.

离子色谱(ion chromatography, IC)是针对离子型物质(或成分)最成熟的分析技术之一。IC不仅可以分析常规无机阴离子和阳离子,还可以分析有机酸、有机胺、糖、氨基酸、草甘膦等大量用HPLC无法或难以分析的强极性有机分子。随着更多基于IC分析方法新标准的不断推出,离子色谱的应用还会进一步普及。强酸或强碱溶液是IC系统最常用的淋洗液,其淋洗离子分别为氢离子和氢氧根离子。由于电解水可产生氢离子或氢氧根离子,因此电渗析器件可在线产生淋洗液用于电致淋洗液发生器(electrodialytic eluent generator, EDG),或产生淋洗液抑制离子用于电致膜抑制器,或用于离子型样品的检测器,或用于离子型样品前处理。围绕这些功能,近年来涌现出一些电渗析新技术。本文将介绍近年来该领域的最新研究动态。

## 1 电致淋洗液发生器

电致淋洗液发生器是一种可在线将纯水转化为酸或碱淋洗液的电渗析器件。相较于人工配制淋洗液,它具有准确度高、重现性好、纯度高(有利于信噪比提升)、自动化程度高等优势;它还能方便地实现IC的梯度淋洗,而且仅需单泵即可产生梯度淋洗,既有利于降低制造成本,也可避免溶液腐蚀泵。

根据结构不同,EDG大致可分为单膜型和双膜型两种类型。单膜型是指只用一种类型的离子交换膜(阴离子交换膜或阳离子交换膜)隔离淋洗液通道和再生液通道,且至少一个电极放置于淋洗液通道内。该器件具有结构简单、淋洗液纯度高等优势,缺陷在于产生的淋洗液含有大量电解气体,须采用脱气装置脱气。该装置相当昂贵,且会导致梯度洗脱时间的延迟。双膜型结构是采用两种类型的离子交换膜(阴离子交换膜和阳离子交换膜)分别隔离淋洗液通道和再生液通道。两电极均放置于再生液通道内,无须脱气。该结构实用化一直面临两个主要瓶颈:一是原材料缺失。阴离子交换膜在阴极区高浓度碱溶液中易被降解(备注:KOH发生器是目前应用最广泛的类型)。目前尚无能满足要求的商品化阴离子交换膜。二是淋洗液纯度低。与单膜结构原位产生淋洗液离子不同,双膜结构是将再生液中的淋洗液离子电迁移至淋洗液通道。在此过程中再生液中的一些杂质离子会不可避免地共迁移进来,从而造成淋洗液纯度低。Lu等^[1]^提出采用双极膜代替阴离子交换膜构建双膜型电致淋洗液发生器的新思路:双极膜的阴离子交换膜面和阳离子交换膜面分别朝向淋洗液通道和再生液通道。因为阴离子交换膜面与阴极区高浓度的碱液不直接接触,故阴离子交换膜不存在降解的可能。目前双极膜已经有多种商品化产品,可以有效解决制造双膜结构EDG的原材料缺失问题;淋洗离子OH^-^来自于双极膜内部界面层增强水解离,而非再生液,故可保证淋洗液的纯度。该双膜型电致淋洗液发生器无须脱气,因此不需要足够高的背压用于辅助脱气,故可用于很多低压环境,如在标准溶液配制、柱后衍生等方面。开管离子色谱(OTIC)因其极低的柱容量和低柱压,很难有兼容的梯度淋洗系统。Chouhan等^[2]^将上述双膜结构拓展用于OTIC,报道了一种纳升级KOH发生器,首次实现了OTIC的梯度淋洗操作。将上述结构稍加变动,即实用双极膜替代上述结构中的阳离子膜,同时使用纯水作为再生液,Sun等^[3]^报道了一种电渗析泵。在电场作用下,来自于阳极区的氢离子和来自于阴极区的氢氧根离子分别电迁移到中间通道,二者可迅速再复合为水,产生的水量(流速)与施加的电流成正比,这事实上为一种新模式的微泵。采用低压电源(<36 V)驱动水溶液可得到纳升或微升级流量,无须特殊的材料或设备,有利于此泵的批量研制,这或为微流控或其他微纳分析技术提供一种新的微泵选择。

碳酸盐淋洗液也常被用于阴离子分析。相比于氢氧根淋洗液,其洗脱能力更强,可在等度洗脱模式下,在较短时间内同时洗脱一价和多价离子,还可以通过改变碳酸氢根和碳酸根比例调控选择性。传统方法制作该类型淋洗液发生器涉及串联酸、碱两个发生器。该方式难以得到高浓度碳酸根淋洗液(比如12 mmol/L)。Shelor等^[4]^报道了一种巧妙的方法解决了此问题。众所周知,液相色谱所用流动相经常涉及脱气处理,脱气原理是管内流动相中的气体在压力驱动下逸出多孔管,对应的装置称之为脱气装置(degasser)。Shelor等^[4]^的方法反其道而行之,让气体靠压差从管外渗透进管内,并溶解在管内的水溶液中。该装置被称之为充气装置(engasser)。具体而言,CO_2_气体在一定压力下渗入到多孔管内的KOH溶液中,二者反应得到K_2_CO_3_溶液。控制多孔管外CO_2_气体的压力即可控制K_2_CO_3_溶液浓度。该方法可得到至少40 mmol/L K_2_CO_3_的淋洗液。该课题组^[5]^将管内KOH溶液替换成纯水,报道了一种与制造可口可乐发生器类似原理的碳酸溶液发生器,即在高压下,CO_2_气体在水中溶解度增大,形成碳酸溶液。该发生器一个直接的应用就是作为阳离子分离用淋洗液。结合弱阳离子色谱柱,该淋洗液可实现碱金属和有机胺等12种离子的基线分离。相对于强酸淋洗液,碳酸淋洗液易分解为纯水和CO_2_,因此可通过降低压力让碳酸淋洗液中的CO_2_逸出,得到近似纯水的淋洗液,可大幅降低电导检测器的背景电流,最终可有效提高系统的信噪比。

相比其他类型的淋洗液发生器,KOH发生器对于人工配制淋洗液方式的优势最为显著,其应用也最为普遍。从技术层面看,发展超高压或低压或不同类型的发生器组合有望成为电致淋洗液发生器未来的主要发展方向。

## 2 电致膜抑制器

抑制器是IC系统区别于HPLC最显著的部件之一,其基本功能是降低淋洗液背景电导、显著提高检测信噪比。电致膜抑制器是最新一代的抑制器,也是IC系统最早商品化的电渗析器件。它是将电导检测器输出的废液电解,得到抑制离子(氢离子或氢氧根离子),而无须再提供人工配制的酸或碱再生溶液。相较于传统的化学抑制器,电致膜抑制器具有绿色、高效、方便等优点,但不足在于存在较高的背景噪声。引起抑制器噪声大的原因很多,但其中一个主要原因在于过量电流的使用。该缺陷在使用 ${\mathrm{CO}}_{3}^{2-}$ 淋洗液时尤其明显。在该淋洗液下,抑制产物碳酸为弱酸,其溶液会存在一个电离平衡,即碳酸溶液会解离成氢离子和碳酸氢根或碳酸根离子。解离出来的H^+^和 ${\mathrm{CO}}_{3}^{2-}$ 的浓度决定了背景电导值。当碳酸浓度不变时,背景电导值理论上亦不会有变化。但由于弱酸多数分子为电中性的特性,碳酸不受离子交换膜唐南(Donnan)排斥影响而扩散至阳极区再生液通道,宏观上造成碳酸浓度变化,最终导致噪声变大。这在淋洗液出口处最为明显,此处碳酸最纯,且阴极再生液通道内的溶液为强碱,会促进碳酸“逃逸”。改善办法就是降低淋洗液出口处的电流。基于此思路,Srinivasan等^[6]^报道了一种基于三电极的电致膜抑制器。阴极分成两部分,之间串联500 Ω电阻。在特定电压下,仅有较小的一部分电压会施加到出口处,可导致较小的背景噪声。它较传统设计可使背景噪声下降约80%。

阳离子电致膜抑制器在循环或外加水模式下会存在峰高变低的现象。究其原因是因为再生液中的 ${\mathrm{HCO}}_{3}^{-}$ 或 ${\mathrm{CO}}_{3}^{2-}$ 迁移到淋洗液通道所致。Zhao等^[7]^提出采用双极膜替代阴极区的阴离子交换膜。抑制酸淋洗液的氢氧根离子来自于双极膜界面层的增强水解离,而不是来自于阴极区的电解水。阴极区通道内的杂质离子无法进入到淋洗液通道内,从而避免了峰高变低的可能。

相对于化学型抑制器,电致膜抑制器最大的不足在于较高的基线噪声。如何降低电致膜抑制器噪声或如何提高电流效率将是未来努力的研究方向。另外,将抑制器作为IC和其他检测器(比如质谱、电感耦合等离子发射光谱等)联用接口将有利于拓宽抑制器的应用范围。

## 3 电渗析样品前处理器

样品前处理的目的主要是提高目标组分的检测灵敏度或消除共存杂质的干扰。基于电场对阴、阳离子反向调控和离子交换膜单向导通性的电渗析技术在处理离子型样品时具有显著优势,可以实现阴、阳离子甚至和中性组分在空间上的自然分离,以净化样品基质。由于6价铬的毒性远高于3价铬,而目前分析技术测定多是总铬含量。利用6价铬在水溶液以阴离子(Cr_2_ $O_{7}^{2-}$ )状态存在、而3价铬以阳离子状态存在的特点,Ohira等^[8]^报道了一种用于处理两种形态铬的电渗析部件,即利用阴离子交换膜、阳离子交换膜,以及两片半透膜制作成相互独立的5通道结构的电渗析器。阴极放置于阴离子交换膜一侧,而阳极放置于阳离子交换膜一侧。阴、阳极所在的通道均位于最外侧。含铬样品进入到电渗析器样品通道后,在电场作用下,3价铬阳离子电迁移至阴极区通道内,与从阴极通道电迁移来的硝酸根结合后进行后续分析;而5价铬阴离子电迁移至阳极区通道内,与从阳极通道电迁移的氢离子结合后进行后续分析;该方法还可拓展用于阴阳离子的在线浓缩^[9,10]^。事实上,该方法理论上还可拓展用于样品的在线除盐,应用于LC-MS或IC-MS系统,但限于半透膜较高的截留相对分子质量,如3.5 kDa,但此相对分子质量仍远远大于小分子的相对分子质量,相对分子质量<500的小分子很容易透过透析膜,因此,中性小分子会有较大的损失。

目前电渗析器件在提高目标离子浓缩倍数上还非常有限,这是目前商品化离子交换膜较高交换容量所致。未来通过对现有商品化离子交换膜进行表面改性,或制备极低交换容量的膜将有助于改善此缺陷。

## 4 展望

电渗析技术与使用强酸或强碱溶液作为淋洗液的IC系统具有很好的兼容性。除了具备上述提到的常见功能外,电渗析技术还有望在标准溶液制备、特殊淋洗液在线制备、弱酸检测等特殊用途上发挥独特作用;伴随着超高压IC、微型化IC的发展,与之匹配的电渗析技术也有必要进行相应的发展。

## References

[1] Lu Y F, Zhou L T, Yang B C, et al. Anal Chem, 2018,90:12840 30274512 10.1021/acs.analchem.8b03365

[2] Chouhan B, Shelor C P, Huang W X, et al. Anal Chem, 2020,92:5561 32138510 10.1021/acs.analchem.0c00505

[3] Sun Y, Lin S Y, Zhang F F, et al. Anal Chem, 2020,92:6263 32295341 10.1021/acs.analchem.0c01077

[4] Shelor C P, Yoshikawa K, Dasgupta P K. Anal Chem, 2017,89:10063 28838239 10.1021/acs.analchem.7b02808

[5] Sricharoen P, Limchoowong N, Shelor C P, et al. Anal Chem, 2019,91:3636 30719920 10.1021/acs.analchem.8b05627

[6] Srinivasan K, Omphroy B K, Lin R, et al. Talanta, 2018,188:152 30029357 10.1016/j.talanta.2018.05.066

[7] Zhao L L, Lu Y F, Zhang F F, et al. J Chromatogr A, 2019,1603:422 31288927 10.1016/j.chroma.2019.06.052

[8] Ohira S I, Nakamura K, Shelor C P, et al. Anal Chem, 2015,87:11575 26507203 10.1021/acs.analchem.5b03464

[9] Ohira S I, Yamasaki T, Koda T, et al. Talanta, 2018,180:176 29332797 10.1016/j.talanta.2017.12.054

[10] Ohira S I, Sakaki T, Miyachi R, et al. Talanta, 2020,216:120989 32456930 10.1016/j.talanta.2020.120989

